# Biomarkers in Skin Mucus for a Minimally Invasive Approach to Stress in Red Tilapia (*Oreochromis* sp.) Fry

**DOI:** 10.3390/biology14020112

**Published:** 2025-01-22

**Authors:** Hernán Antonio Alzate-Díaz, Laura Fernández-Alacid, Sandra Clemencia Pardo-Carrasco

**Affiliations:** 1 Department of Animal Production, Faculty of Agrarian Sciences, Universidad Nacional de Colombia, Medellín Campus, Carrera 65 #59ª-110, Bloque 50 Of. 309 Nucleo El Volador, Medellín 050034, Colombia; haalzated@unal.edu.co; 2 Department of Cell Biology, Physiology and Immunology, Faculty of Biology, University of Barcelona, Avda. Diagonal 643, 08028 Barcelona, Spain; fernandez_alacid@ub.edu

**Keywords:** skin mucus, welfare, biofloc, red tilapia (*Oreochromis* sp.)

## Abstract

In a context where aquaculture continues to grow, it is essential to understand how culture conditions influence fish stress. The welfare of red tilapia fry (*Oreochromis* sp.), weighing 0.6 ± 0.3 g and with an initial total length of 3.548 ± 0.285 cm, was evaluated in two culture systems, biofloc and land-based ponds, over a period of 30 days through biomarkers associated with the skin mucosa, such as protein, glucose, lactate, and the hormone cortisol, in addition to their relationships with protein, seeking a comprehensive view of the physiological state and stress of these fish. Mucus samples were thawed and prepared by mechanical homogenization and centrifugation. The results reveal that the biofloc system provides a more conducive environment for welfare, evidenced by higher mucus protein levels of 1.598 ± 0.733 mg/mL, 2.316 ± 1.01 mg/mL, and 1.203 ± 0.640 mg/mL for culture days 10, 20 and 30, respectively, and lower cortisol > 1 ng/mL and lactate contents 1799 ± 1135 µg/mL, 30,271 ± 24,613 µg/mL, and 21,829 ± 23,507 µg/mL for culture days 10, 20 and 30, respectively, compounds largely associated with animal stress. This highlights the direct relationship between culture, environment and fish health and highlights skin mucus as a valuable minimally invasive biomarker. It is also important to note that improved fish welfare supports fish growth and health and promotes more sustainable aquaculture practices, contributing to more efficient and responsible production in the industry.

## Simple Summary

Today, fish cultivation is continuing to grow, so it is essential to understand how their culture conditions influence their health. We assessed the welfare of red tilapia fry in two culture systems, (1) biofloc (a sustainable aquaculture practice that uses microbial communities, known as bioflocs, to maintain water quality and provide supplemental nutrition for cultured fish) and (2) traditional ponds, for 30 days, employing skin mucus-associated biological markers, including protein, glucose, lactate, and the hormone cortisol, and determining their relationships with protein. We used this methodology to obtain a comprehensive view of the physiological state and welfare of the fish cultured. The results revealed that the biofloc system offers a more favorable environment for fish welfare, evidenced by registering higher protein levels in the mucus and lower contents of cortisol and lactate, compounds associated with animal stress. These results emphasize the direct relationship between the culture environment and the fish’s health and highlight skin mucus as a valuable biomarker. It is also important to note that by improving fish welfare, their growth and health will also improve, and we can promote more sustainable aquaculture practices in this way, contributing to more efficient and responsible large-scale fish production.

## 1. Introduction

The production and consumption of aquaculture products are continuously expanding. According to the FAO report of 2024 [1], aquaculture has grown faster (51%) than capture fisheries, which have been surpassed for the first time. In the same way, as production and technical development in aquaculture have grown, interest in the welfare of fish has grown [2]. Welfare becomes important in production due to issues such as product acceptance, marketing, and production efficiency beyond public perception of management, which mainly generates pressure on intensive production [2,3].

The concept of welfare applied to fish has not been proposed for a long time, although there is scientific evidence that, like other vertebrates, fish respond to stressful stimuli of pain and fear. Conditions of low or deficient welfare are manifested when fish are subjected to situations of pain, fear, and lack of control over their environment or if they are sick, injured, or hungry [3]. Discussing welfare in fish requires the evaluation of the stress response, an adaptive function to a stimulus from the environment that can be short and allow, among many aspects, homeostasis to be maintained, prolonged or chronically, and this will determine whether or not it is harmful to the animal in terms of decreased welfare or the feeling of suffering [2]. This is why, in productive terms with a welfare approach in aquaculture, emphasis has been placed on avoiding the unadapted consequences of prolonged stress that can generate adverse effects on the animal, such as decreased immune response and growth, and suppression of its reproductive function [2,4,5].

At a physiological level, the brain–sympathetic–chromaffin cell axis is a critical component of the stress response in fish, enabling them to react swiftly to environmental challenges. This axis involves a complex interplay between the brain, the sympathetic nervous system, and chromaffin cells, which are specialized neuroendocrine cells [6]. Also, the response to stress in teleost fish involves the activation of the hypothalamic–pituitary–interrenal (HPI) axis, triggering the hypothalamic reaction from the action of the corticotropin-releasing factor (CRH) in the pituitary gland, which in turn releases the adrenocorticotropic hormone (ACTH) that allows the release of the glucocorticoid stress hormone called cortisol by the interrenal cells, generating secondary or tertiary stress responses, or both [2,7]. The activation of the HPI axis causes the animal to mobilize its energy reserve in the form of glycogen and increase plasma glucose levels. In addition, muscular activity causes anaerobic glycolysis and an increase in plasma lactate concentration [7,8]. Therefore, stress in teleosts is assessed by the concentrations of cortisol, glucose, and lactate present [7].

The measurement of welfare conditions such as stress in fish has been carried out conventionally with chemical hematological tests of metabolites and hormones [9]. These tests allow the evaluation of the state of welfare, but there is excessive body contact and invasion with the use of needles. Therefore, exploring minimally invasive techniques, e.g., by interpreting the composition of skin mucus, can be comparable to working with classical techniques [9,10,11,12,13,14]. In a study carried out by Fernández-Alacid et al. [11], the authors compared mucus and plasma samples from (*Argyrosomus regius*) under pre- and post-stress conditions due to management conditions. The levels of glucose, lactate, proteins, and cortisol were determined, and correlations were established between stress metabolites in plasma and mucus that allowed a comparison of the levels of these biomarkers in plasma and mucus under different conditions, which helped the researchers to better understand the response dynamics and the effectiveness of using skin mucus as a stress-monitoring tool. Other authors, such as Santoso et al. [15] and Carbajal et al. [12] with (*Oncorhynchus mykiss*), reached the same conclusion when they analyzed these biomarkers in invasive and minimally invasive methods.

Due to its lipophilic composition, concentrations of cortisol—the stress hormone—are mobilized from the blood to the epidermal tissue, and the results are correlatable [11]. It is essential to mention that, as Fernández-Alacid et al. [16] stated, studies should be performed on different species, finding reference values in animals under normal (control data) and stress conditions. It is also important to note, as mentioned by Sanahuja et al. [13], that the extraction of skin mucus after single or multiple events has proven to be as effective as a minimally invasive methodology at the epidermis level, without affecting the integrity of the skin nor its barrier properties compared to the intact skin of non-sampled fish.

The mucus of fish skin, among its multiple functions, stands out for functioning as an external barrier against microorganisms in a non-specific way, with a variable composition between different fish species [17,18,19]. The composition of the skin mucus is heterogeneous and influenced by endogenous factors, such as sex, stage of development, and other exogenous factors, including hyperosmolarity, water pH, infections, and stress [20]. It contains compounds of innate immunity, mainly of protein origin [21,22], such as lysozymes with bacteriolytic power, lectins, proteases, and antimicrobial peptides, with proven action against pathogens [19,23]. According to Birchenough et al. [24], goblet cells release abundant glycoproteins (called mucins) that form the mucus layer, which is mediated by the interaction of molecules such as acetylcholine and histamine and allows the protection of the intestinal mucosa. This reaction is also present in the skin mucosa. The skin mucus can be viewed from different approaches; at the physical level, it is considered a viscous colloid, and at a biological level, it is recognized as a composition of glycoproteins called mucins, in addition to other compounds such as carbohydrates, water, and antimicrobial agents, among others [19]. In the proteome of the skin mucus, nearly 100 different proteins and 12 functional groups have been identified, and they are also classified as structural, metabolic, and protection proteins [21]. Likewise, skin mucus has protection, locomotion, respiration, ionic, and osmotic regulation, and excretion functions, among others [19]. Among the identified proteins, enzymes with biocidal action, such as lysozymes, phosphatases, proteases, esterases, and cathepsins, are found [21,22].

Only the soluble fraction of the content is used to determine animal welfare in fish through the skin mucus evaluation in which key markers recognized as “skin mucus associated biomarkers” (SMABs) have been identified, among which cortisol, lactate, soluble protein, and glucose can be highlighted as the most important for this determination and of which certain relationships or ratios, such as glucose/soluble protein, lactate/protein, and cortisol/protein, among others, can be analyzed [16,25].

Likewise, due to the variation in mucus composition between different fish species, it is necessary to determine its composition in individuals of productive importance, such as the red tilapia hybrid (*Oreochromis* sp.). In Colombia, genetic studies in red tilapia indicate that introgression between fish farms is significant for (*O. aureus*), while the species (*O. niloticus*) and (*O. mosambicus*) are similarly introgressed in populations of *Oreochromis* sp. [26]. On the other hand, high mortalities are reported in the fry stage. For this reason, new fry-rearing systems are being attempted, such as BFT, which, being intensive, allows for greater control and water and space savings, compared to the traditional, semi-intensive system in earthen ponds, with low water exchange. Additionally, according to some scientific articles, despite the high density, biofloc provides benefits to tilapia cultivation in resistance to pathogens, immunity, and growth [27,28,29]. Accordingly, the aim of this work was to establish the baseline values of biomarkers associated with the skin mucus in a minimally invasive way and to compare the stress levels of red tilapia fry in both systems over a 30-day period.

## 2. Materials and Methods

### 2.1. Water Quality Parameters and Experimental Conditions

During the fry growth period, dissolved oxygen (DO), pH, temperature, and total dissolved solids (TDSs) were measured twice a day with a Hanna HI98494 multiparameter probe. Additionally, ammonium, nitrite, nitrate, and alkalinity were measured every three days with a YSI 9500 photometer. Specifically, for the BFT system, total suspended solids (TSSs) were measured with an Imhoff cone every three days (Table 1).

Red tilapia (*Oreochromis* sp.) were acquired from a farm near the experimental area, caught, fasted for 24 h, packed in bags with water and oxygen, sealed, and transported to the experimental farm (less than 3 h of travel). Upon arrival, the bags were distributed in the experimental units, left closed and floating for acclimatization for 30 min, and then opened, mixed with the water of the experimental unit, and finally released. The fry weighing 0.6 ± 0.3 g and initial total length of 3.548 ± 0.285 cm were cultured for 30 days in two production systems, biofloc technology (BFT) and land-based ponds (POND). The ponds (four experimental units) had a volume of 24 m^3^ located outdoors, a stocking rate of 25 fish/m^3^, and controlled water entry and meshes to prevent the entry of predators. The biofloc tanks (four experimental units), each of 1 m^3^ and with a stocking rate of 400 fish/m^3^ (Table 2), were located indoors, and a C/N ratio of 20/1 to encourage the heterotrophic bacterial culture was managed, following the requirements of Alzate-Díaz et al. [30] and Emerenciano et al. [31], adding cane molasses as a carbon source. The aeration of the tanks was performed with a pump (HG-C/C2, 1/4 HP, Pump Power^®^, Jacksonville, FL, USA), to which a system of micro-perforated air diffuser hoses was attached from the bottom of the tank in order to favor the resuspension of the particles; the total suspended solids were measured with Imhoff cones. The biofloc preparation started with red Californian earthworm (*Eisenia foetida*) leachate at a rate of 1 L per tank. The units were left for 30 days to mature with aeration, and during this time, molasses at a rate of 0.02 g/L was used as a carbon source, and ammonium chloride (NH_4_Cl) at 5 mg/L, sodium bicarbonate (NaHCO_3_) at 50 mg/L, and sea salt at 2 g/L were added daily to the water. The fry were fed a commercial diet of 45% crude protein, dry matter 88%, lipid 6%, fiber 4%, and ash 12% [32], six times a day at a rate of 8% of live weight.

### 2.2. Productive Performance

At each mucus sampling, the fish were weighed and measured. Mortality and the amount of food consumed were recorded daily. At the end of the 30 days of culture, the daily weight gain (DWG), feed conversion rate (FCR), specific growth rate (SGR), survival (%), and final biomass were determined (g/m^3^).

DWG = (fW − iW)/(t); SGR = 100 × [(lnfW − lniW)/(t2 − t1)]; FCR = (F/WG); K = 100 × W/L^3^; Survival = 100 × (fN/iN).

where iW = initial weight; fW = final weight; ln = natural logarithm; t = time; WG = weight gained (g); F = food (g); W = weight; L = length; fN = final number of fish; iN = initial number of fish.

### 2.3. Sampling of Skin Mucus

Skin mucus was obtained on days 10, 20, and 30 of the culture from the POND and BFT experimental units. The fish were anesthetized with eugenol at a dose of 0.2 mL/L of water; after losing the swimming axis, they were taken out of the water, and mucus was collected by scraping the surface of the epidermis between the thoracic and abdominal areas above the midline of the individual. At each time and from each experimental unit (4 BFT and 4 POND), 25 fry were sampled to complete the amount of mucus necessary for the analyses. On each culture day (3), 8 samples were taken (2 treatments with 4 replicates) and each one was divided into three aliquots for analysis, for a total of 72 samples analyzed. The sampled fry were removed from the experiment. Mucus was extracted using an adaptation of the technique proposed by Fernández-Alacid et al. [16]. The samples were preserved in liquid nitrogen and then transferred to dry ice to facilitate shipment to the Department of Cellular Biology, Physiology and Immunology of the Faculty of Biology of Universidad de Barcelona (Spain).

### 2.4. Characterization of Skin Mucus

The mucus was thawed and prepared through mechanical homogenization using a Pistón Pellet Eppendorf, and centrifuged in Eppendorf 5418R equipment for reading biomarkers. The aqueous phase was used to evaluate the biomarkers in mucus cortisol, glucose, lactate, and protein. Cortisol was measured using a competitive Elisa kit (ELISA kit, IBL International, Hamburg, Germany. Ref. 52611). Soluble glucose concentration was determined through an enzymatic colorimetric test (LO-POD glucose, SPINREACT^®^, Girona, Spain. Ref. MX41011); soluble lactate concentration was measured with an LO-POD lactate analysis (SPINREACT^®^, Girona, Spain. Ref. 1001330), and protein concentration was measured by the Bradford method (BSA; Sigma, St. Louis, MO, USA) (Bradford, 1976). All readings were performed in triplicate on the Tecan Nanoquant Infinite N200 equipment adapted for mucus, according to Fernández-Alacid et al. [16].

### 2.5. Statistical Analysis

For the variables of importance, descriptive statistics (mean, median, standard deviation, minimum and maximum value, and number of data) were estimated in general and discriminated by treatment.

For the inferential analyses, the following linear model was used:

$$Y_{ij}=\mu +\tau_{j}+Cov\left(dc\right)+\varepsilon_{ij}$$

where $Y_{ij}$ is the response variable or variable of importance in the study, μ is the general mean of the response variable, $\tau_{j}$ is the treatment factor where j = 2 (Treatment 1 = Pond; Treatment 2 = Biofloc), $Cov\left(dc\right)$ is the covariate culture day, and $\varepsilon_{ij}$ is the experimental error. It is worth mentioning that when evaluating the culture days as a covariate in the statistical model, there was no impact on the SMABs as the evaluated treatments did. To validate all inferential analyses, the assumptions of normality and homoscedasticity of the residuals were validated: ε ~ N (0, σ_e^2^). The analyses and figures were carried out using the programs ggplot2, tidyverse, corrplot, FactoMiner, and factoextra of the specialized software R Project version 4.4.0 [33].

**Table 1:** Physicochemical parameters of water quality in the experimental units.

Parameter	POND	BFT
Temperature (°C)	28.68 ± 1.119 a	26.32 ± 0.628 b
pH	7.63 ± 1034 a	7.414 ± 0.706 a
DO (mg/L)	3.58 ± 1413 b	7.65 ± 0.649 a
Alkalinity (mg/L)	70.142 ± 19,231 b	98.878 ± 33.767 a

Data correspond to the mean of the replicates ± SD. Different letters (a, b) suggest statistically significant differences.

**Table 2:** Results of productive performance of *Oreochromis* sp. fry during cultivation time.

Indicator	POND	BFT
Stocking density (fry/m^3^)	24	400
Replicates (Unit)	4	4
Stocking fish by unit	600	400
Initial total biomass (g/m^3^)	16.07± 4.45 a	257.98 ± 72 b
Final total biomass (g/m^3^)	87.887 ± 31.020 a	1069.045 ± 192.268 b
DWG (g/day)	0.251 ± 0.186 a	0.222 ± 0.189 a
SGR (%/day)	8.449 ± 3.024 a	7.948 ± 2.396 a
FCR	1.12 ± 0.56 a	1.30 ± 0.47 a
Survival (%)	87.80 ± 13.31 a	81.74 ± 10.42 a
K	1.072 ± 0.089 b	1.154 ± 0.0 a

DWG: daily weight gain; SGR: specific growth rate; FCR: food conversion rate. K: condition factor. Data correspond to the mean of the replicates ± SD. Different letters (a, b) suggest statistically significant differences.

## 3. Results and Discussion

### 3.1. Water Quality

The water quality parameters for the two treatments presented optimal conditions and within the comfort ranges for the evaluated productive (*Oreochromis* sp.) stage (Table 1). The oxygen in the ponds is lower, but the biomass is very low, and during the study, no respiratory distress was evident at any time.

### 3.2. Productive Performance of Oreochromis sp. Fry

The results of water quality and productive performance of the fry (Table 2) show that the conditions in the two systems were satisfactory; however, the fish cultured in BFT would have a greater possibility of performing in the face of challenging conditions, since they were evidently less stressed. Even though the productive performance had no difference between the systems, the BFT system is intensive and allows for greater production of fry per liter of water and space, and yet, they are in a better state of welfare.

### 3.3. Biomarkers Evaluated in Skin Mucus

The results of the stress biomarkers in the two evaluated systems were as follows:

The amount of total protein in skin mucus is influenced by factors such as fish species, temperature, different ecological niches, mucus collection methods, and different stages of development [34]. It should be noted that the concentration of soluble protein in skin mucus is evaluated as a biological marker of the physiological state of teleosts; this is because the composition allows different physiological functions, such as structural, enzymatic including proteases and esterases, and immunological including lysozymes. For this reason, the protein concentration of skin mucus determines changes in the physiological state of the fish and at the physicochemical level if it is a concentrated or diluted mucus [23,25]. Similarly, soluble protein content is also recognized as a reference biomarker for homeostatic effects on fish mucus [25].

**Figure 1:**
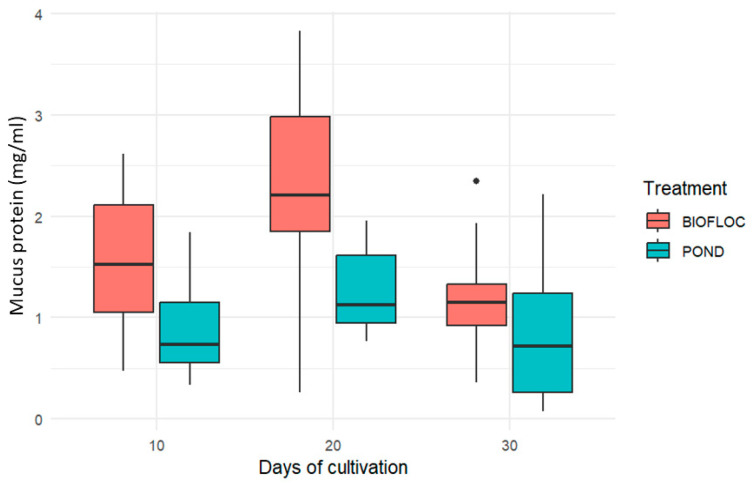
Soluble protein content in the skin mucus of *Oreochromis* sp. in two culture systems.

As can be observed in Figure 1, the soluble protein content in the mucus of individuals cultured in BFT presents values of 1.598 ± 0.733 mg/mL, 2.316 ± 1.01 mg/mL, and 1.203 ± 0.640 mg/mL for days 10, 20 and 30, respectively. A significant difference between BFT and POND was only registered on day 20 of the culture. In different studies carried out, it has been possible to relate the skin mucus to higher protein contents that provide fish with greater protection against stress and infections by pathogens that may put their health at risk [11,21,35]. Likewise, regarding the proteome of the skin mucus, by monitoring the presence and expression of some proteins in the mucus, information can be obtained on the physiological state of the fish in a minimally invasive way [21]. Baba [34] conducted an experiment in which he measured the protein in the skin mucus of four juvenile Nile tilapia (*Oreochromis niloticus*). In these results, he found protein contents for *O. niloticus* of 11.59 ± 0.38 mg/mL. The difference with the present study may be related to the weight, age, and species of the fish. Finally, it is important to state that biofloc systems and their contributions to microbial protein confer an immunostimulating action, which improves the concentration of proteins related to immunity, as reported by Debbarma et al. [35] for the case of albumin and globulin.

Different studies have shown that glucose is a commonly used indicator in fish research as a response to stress and health status [36] and that it is a crucial carbohydrate in the bioenergetic processes of fish [11]. Changes in glucose levels in skin mucus are associated with physiological responses to stress, activation of the interrenal axis, and the release of hormones such as cortisol, which can affect the metabolic conditions of fish [36]. It is important to note that the mobilization and increase in glucose are mainly due to the energy demand given by processes such as gluconeogenesis and glycogenolysis to meet the energy demand [11] in stress situations received by fish during their cultivation period [23,36,37].

In a study carried out by Fernández-Alacid et al. [11], it was established that glucose concentrations were directly related to the increase in cortisol, in accordance with what was found in the present study.

**Figure 2:**
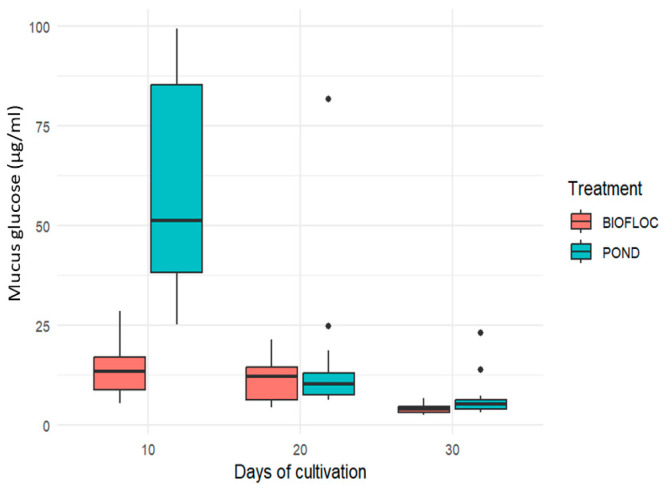
Glucose content of the skin mucus of *Oreochromis* sp. in two culture systems.

Figure 2 shows the glucose contents in skin mucus for *Oreochromis* sp., where it can be established that for 10 days of cultivation, there was a significant difference (*p* < 0.05) between the treatments. Thus, a concentration of glucose in the ponds is notable, with 58.267 ± 27.16 µg/mL compared to bioflocs, which, when analyzed with the results registered for cortisol of this research, show a direct relationship with this hormone, and higher values in the ponds for glucose and cortisol in the same cultivation time. Subsequently, the glucose and cortisol levels recorded in the ponds at 20 and 30 days of culture decreased, and in bioflocs, there was no major variation. However, it remained in decline without significant differences between the two treatments (*p* > 0.05), presenting values of 17.025 ± 2.06 µg/mL and 7.123 ± 5.74 µg/mL for the ponds, and 11.959 ± 6.202 µg/mL and 4.183 ± 1.626 µg/mL for bioflocs, allowing us to clearly observe a lower glucose content in the latter throughout the culture time.

In the studies conducted by Debbarma et al. [35], the glucose contents in bioflocs were noticeably lower compared to the control treatment in the moments after being subjected to stress caused by ammonium in almost all the C/N ratios evaluated, allowing us to determine that exposure to a stressor increases glucose levels, which is common in these situations [35].

Soluble lactate allows us to identify the state of the anaerobic energy metabolism of fish [36]. This metabolic product is produced by epidermal cells, among others, and can accumulate in tissues during stress or intense exercise situations [11]. Its high concentrations in skin mucus can indicate acute or chronic stress and, therefore, it is necessary to monitor this biomarker to evaluate the state of welfare and health of fish, determining whether it is a product of typical situations of increased activity or intense exercise by the animal or adverse situations such as subjection to periods of hypoxia or other reasons [7,23,36]. More precisely, Fernández-Alacid et al. [11] mention that lactate reacts quickly to acute stress situations such as hypoxia [13] and capture or to other changes such as salinity [25] and high concentrations of ammonium [35]. It increased rapidly, but its concentration was not sustained and decreased over time, demonstrating that it can be a good biomarker to measure an immediate stress response, but not necessarily for long-term situations.

**Figure 3:**
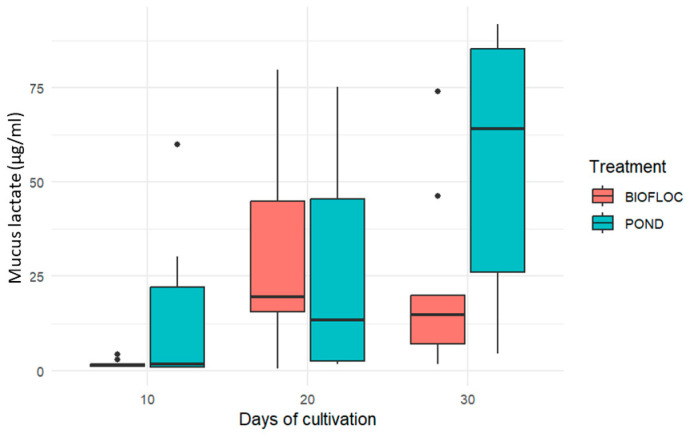
Lactate content of the skin mucus of *Oreochromis* sp. in two culture systems.

The lactate values obtained can be seen in Figure 3. There was only a significant difference between the treatments at day 30 of the culture (*p* < 0.05). In the fish cultured in ponds, there was a gradual increase in lactate, which went from having a value of 25.890 ± 27.37 µg/mL on day 20 of the culture to 55.83 ± 24.61 µg/mL on day 30 of the culture. On the other hand, in the biofloc culture, the values were lower at the beginning, i.e., on day 10 with 1.799 ± 1.135 µg/mL, increasing on day 20, and finally showing values lower than those reported for ponds of 21.829 ± 23.507 µg/mL. The average values of soluble lactate were very variable, as Fernández-Alacid et al. [16] also reported.

Cortisol is undoubtedly the most related hormone when discussing acute or critical stressful situations in different vertebrates, including fish [3,9]. Under natural conditions, this hormone acts adaptively to management conditions such as high densities [38], hypoxia [11], and capture [8], and environmental conditions such as changes in salinity and energy metabolism [7,25] and increased ammonium [35], among others. Likewise, at a physiological level, it acts as a hormone mediating the regulation of functions such as metabolism, osmoregulation, and immune response. This hormone, which belongs to the glucocorticoid group, is released in response to the stimulus of the hypothalamus–pituitary–interrenal (HPI) pathway, which, as a chain reaction, stimulates the release of the metabolite glucose that is absorbed and lactate that accumulates in the tissues [7].

In stressful situations, fish generate an increase in the concentration of cortisol in the skin mucus; in mild and acute conditions, they generate an increase in cortisol in a time span of 30 to 60 min, and its reduction can last between 3 and 4 h from the beginning of the stressor [9]. In a study carried out by Fernández-Alacid et al. [11], the authors found that under stressful hypoxia conditions, cortisol contents return to their basal levels 6 h after exposure, which can be seen as an effective homeostasis process. This reduction in cortisol over time is due to cortisol catabolism, where the hormone undergoes conversion to metabolites with lower activity, resulting in a decrease in cortisol concentrations in the individual, a conservation response to avoid adverse effects due to elevated levels of the hormone [10].

Cortisol concentrations have been found to be quite variable among different fish species [16]. Conversely, when cortisol is analyzed through plasma and skin mucus, these show a positive correlation, establishing that skin mucus is a good minimally invasive biomarker for the acute response to stress [11,13], providing information on the physiological state of the fish without causing additional stress, which is an important aspect for fish production [23,36].

**Figure 4:**
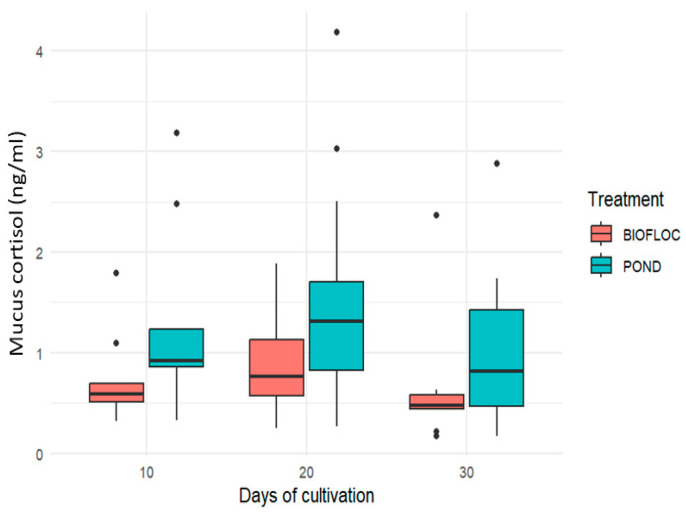
Cortisol content of the skin mucus of *Oreochromis* sp. in two culture systems.

In Figure 4, the concentration of cortisol in the skin mucus of *Oreochromis* sp. under different treatments is presented, and no significant difference was registered (*p* > 0.05) in any of the analyzed culture periods. Cortisol values in the pond treatment are 1.253 ± 0.957 ng/mL, 1.534 ± 1.152 ng/mL, and 1.027 ± 0.768 ng/mL for culture days 10, 20, and 30, respectively, values that did not exceed 1 ng/mL for biofloc in these culture times. In a study carried out in a biofloc system with *O. niloticus* by Azim and Little [39], in which plasma cortisol was evaluated, these authors found a higher concentration of cortisol in this system when it was related to the control treatment consisting of a recirculating aquaculture system (RAS), stating a greater challenge due to the bacterial load and the physical dynamics presented by the biofloc system. In another study carried out by Debbarma et al. [35], the authors evaluated the plasma cortisol of *O. niloticus* in biofloc systems with ammonium as a stressor under different C/N ratios. In this study, the authors managed to identify that high post-stress concentrations were present in the 10/1 ratios and lower concentrations in the 15/1 and 20/1 ratios. In the current study, a 20/1 ratio was used, validating that it is an adequate ratio for cultivating this species, contributing to lower cortisol concentrations in the system.

The ratios of metabolites (glucose, lactate, and cortisol) to protein values of skin mucus are presented below to correct for possible dilution of the mucus at the time of collection.

**Figure 5:**
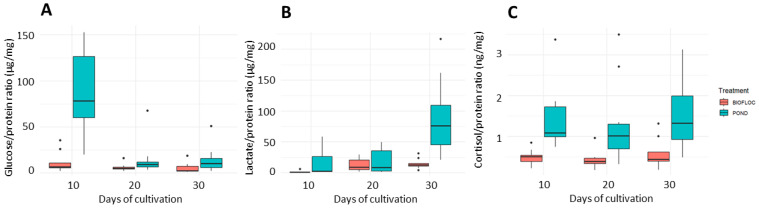
Glucose/protein (**A**), lactate/protein (**B**), and cortisol/protein (**C**) ratios of skin mucus of *Oreochromis* sp.

In Figure 5A, corresponding to the glucose/protein ratio, significant differences occurred on day 10 of the cultivation, where the highest values are related to the pond treatment. However, these values decrease for the subsequent periods evaluated, showing an adaptive process to the system, which differs from the behavior of the biofloc that maintains a low ratio throughout the cultivation time. The glucose/protein ratio is identified as a reliable biomarker of energy metabolism, being the most suitable for reflecting the skin response [16] and for monitoring physiological responses such as environmental or anthropogenic challenges [13]. This situation is mainly due to the mobilization of glucose by metabolic pathways to meet the energy demands that occur in the defense and escape reactions of fish.

For Figure 5B, the lactate/protein ratio shows significant differences for the 30 days of culture, where the pond treatment presents the highest values. It is essential to mention that, as Fernández-Alacid et al. [16] stated, high lactate levels combined with decreased mucus protein are considered a response to acute stress that causes deterioration of fish health. It is also evident that, as shown in the glucose/protein ratio, the biofloc behaves with lower values in this ratio, and therefore, lower metabolic wear in the biofloc system can be distinguished and, hence, better welfare conditions. The lactate/protein ratio measured in skin mucus is considered a relevant biomarker of stress in fish, providing valuable information on their physiological state under adverse conditions [11].

Finally, Figure 5C shows the cortisol/protein ratio, showing significant differences between the two treatments. Higher cortisol values were presented in the fish from the ponds compared to those cultivated in biofloc. This can clearly explain that the biofloc system provided a more suitable environment due to the lower presence of cortisol throughout the cultivation time expressed through this cortisol/protein ratio. This ratio allows the health and welfare status of the fish to be established conclusively, and it can also be identified that the skin mucus not only acts as an immunological barrier but also reflects the physiological state of the fish under culture conditions [25]. On the other hand, the cortisol/protein ratio provides a corrective action on the skin mucus, allowing cortisol values to be normalized and giving greater reliability. Through this ratio, cortisol values are expressed concerning the amount of protein present, allowing the attainment of a more precise measure of the stress status of the fish, regardless of the dilution with water that may have occurred during sample collection [16,25].

## 4. Conclusions

Skin mucus-associated biomarkers (SMABs) are reliable for measuring stress in red tilapia (*Oreochromis* sp.) fry in a minimally invasive way, which is crucial to improving management practices in aquaculture.

The results indicate that the biofloc system could present better conditions for the cultivation of red tilapia fry, but further studies are needed under different conditions.

The cortisol/protein ratio allows us to conclude that fry raised in BFT had lower stress levels, allowing the production of tilapia fry in a system that has been identified as beneficial for fish.

## Data Availability

The data that support the findings presented in this study are available in “Repositorio UNIVERSIDAD NACIONAL DE COLOMBIA” upon direct request to the corresponding author.
